# Ozonation as an effective way to stabilize new kinds of fermentation media used in biotechnological production of liquid fuel additives

**DOI:** 10.1186/s13068-016-0574-2

**Published:** 2016-07-22

**Authors:** Piotr Dziugan, Maria Balcerek, Michal J. Binczarski, Dorota Kregiel, Marcin Kucner, Alina Kunicka-Styczynska, Katarzyna Pielech-Przybylska, Krzysztof Smigielski, Izabela A. Witonska

**Affiliations:** Faculty of Biotechnology and Food Sciences, Institute of Fermentation Technology and Microbiology, Lodz University of Technology, Wolczanska 171-173, 90-924 Lodz, Poland; Faculty of Chemistry, Institute of General and Ecological Chemistry, Lodz University of Technology, Zeromskiego 116, 90-924 Lodz, Poland; Faculty of Biotechnology and Food Sciences, Institute of Technology and Food Analysis, Lodz University of Technology, Wolczanska 171-173, 90-924 Lodz, Poland; Faculty of Biotechnology and Food Sciences, Institute of Food Chemistry, Lodz University of Technology, Wolczanska 171-173, 90-924 Lodz, Poland

**Keywords:** Ozonation, Raw sugar beet juice, Sterilization, Bioethanol production

## Abstract

**Background:**

Intermediates from processing sugar beets are considered an attractive feedstock for ethanol fermentation due to their high fermentable sugar content. In particular, medium prepared from raw sugar beet juice seems to be suitable for use in fermentation processes, but it is microbiologically unstable and requires sterilization.

**Results:**

This study investigates the effect of ozone treatment on the activity of microbial cells from *Bacillus subtilis,Leuconostoc mesenteroides*, *Geobacillus stearothermophilus*, *Candida vini,* and *Aspergillus brasiliensis* in raw sugar beet juice. Raw sugar beet juice contaminated with 10^5^ cfu/mL of the microbial strains was treated with gaseous ozone (ozone concentration in the oxygen stream 0.1 g O_3_/L O_2_, flow rate 6 L/h, 10–30 min, 18–20 °C). The number of microflora decreased to 0 cfu/mL after 30 min of ozone treatment in all studied samples.

**Conclusions:**

Medium prepared from raw sugar beet juice and sterilized by ozonation is suitable for use in fermentation processes.

## Background

Ethanol fermented from media obtained through hydrolysis of starchy or lignocellulosic biomass has great potential as a renewable and sustainable energy source. Unfortunately, although the process of producing ethanol from sugar is well established, it is still nearly twice as expensive as producing gasoline from crude oil. Hydrolysis of starchy or lignocellulosic biomass to yield fermentable sugars is also still too costly and difficult to implement on a large scale [22, 37]. However, less expensive bioethanol can be produced directly from the juices of free sugar containing crops, such as sugarcane juices, sugar beet juices, sweet sorghum juices, and some fruit juices [10]. Sucrose, which is the main sugar in fermentable juices, is readily broken down into glucose and fructose during the early stage of fermentation by invertase in the periplasmic space of yeast cells [7]. Vučurović et al. [36] performed a sensitivity study to examine the costs of sugar beet and yeast in ethanol production. They calculated capital investment costs, unit production costs, and operating costs for a plant producing 44 million L of 99.6 % pure ethanol annually. The results clearly demonstrate that raw material costs have a significant impact on the expense of producing ethanol.

Intermediates from processing sugar beets, which grow well in the Polish climate, are considered an attractive feedstock for biofuel fermentation due to their high fermentable sugar content. Moreover, sugar beet juices (raw juice as well as thin and thick juices) do not require enzymatic treatment, unlike raw materials that contain starch. Raw juice may be fermented to produce ethanol with a good yield (85–87 g/L) after supplementing with mineral salts [13]. Thick juice has the additional advantage of high solid substance and saccharose content, which eliminates storage issues. This feedstock can be used for the fermentation of bioethanol [8] and biobutanol [9] or valuable chemicals, such as propylene glycol [4]. A method of producing bioethanol from thick sugar beet juice is described in detail in a previous publication by Dziugan et al. [8].

With all sugar beet juices (raw, thin, and thick juices), we detected microbial contamination in the fermentation broths, which affected the yield of bioethanol. The conventional methods of thermal sterilization of fermentation media are an energy-intensive process and require special equipment. On the other hand, the application of antibiotics in fuel ethanol production is controversial. There are two major concerns with regard to the use of antibiotics. First is the potential for bacteria to develop resistance, rendering antibiotics ineffective against infections [20]. Second, there is concern over the potential for antibiotic residues to remain in animal feeds (i.e., DDGS—Dried Distillers Grains with Solubles) and potentially in animal tissues destined for human consumption [3]. For these reasons, we decided to develop a simple method of sterilizing sugar beet juices using ozone. Because ozone has a high oxidation potential in alkaline solution (2.07 V) compared to chlorine (1.36 V), it can be an effective antimicrobial agent [12, 16]. In 2001, ozone in gaseous and aqueous phases was approved by the US FDA as an antimicrobial agent to be used in the treatment, storage, and processing of foods [15]. Since then, this triatomic allotrope of oxygen has been used in many industrial processes, including water purification (for drinking water, bottled water), soft drink manufacturing, the production and processing of fruits and vegetables, and the processing of fish and seafood, as well as for the hygienization of equipment used in the food industry [5].

Ozone is safer than many other chemical disinfectants, including ethylene oxide, chloride, chlorine dioxide, sodium hypochlorite, isopropyl alcohol, phenolics, and iodophors, because it has a much shorter half-life than such agents, and the only product left over from ozone disinfection is oxygen. Ozone can also remove color strains and odors more effectively and destroy all forms of microorganisms at relatively low concentrations, which is particularly important for the food industry. Several studies have described the antimicrobial action of ozone treatment against a wide range of microorganisms in apple juice [6, 24, 28, 29, 33, 34], orange juice [23], strawberry juice [30], grape juice [31], and blackberry juice [32]. These studies demonstrate that more than 5-log reduction in pathogenic microorganisms can be achieved in fruit juices using ozone.

Because of its oxidizing properties, ozone is considered one of the fastest and most efficient known microbicides. However, the mechanism by which ozone inactivates microbes is still not properly understood. In aqueous solution, ozone may react with microbes either indirectly, with the radical species formed when ozone decomposes, or directly with molecular ozone. Ozone is known to attack unsaturated bonds, forming aldehydes, ketones, or carbonyl compounds. It can break down cell membrane or protoplasm, inhibiting cellular reactivation of bacteria, coli forms, viruses, and protozoa. At 10 mg/L, it removes up to 99 % of bacteria and viruses in 10 min, attacking mainly unsaturated fatty acids, lipid fatty acids, glycoproteins, glycolipids, amino acids, and sulfhydryl groups of some enzymes. The resistance mechanism of spores makes them very difficult to combat, with generally useful treatments, such as high temperatures and the use of antimicrobial agents becoming ineffective. Ozone at concentrations slightly higher than those used for other bacteria can overcome spore resistance [26].

There are no reports in the literature on the use of ozone to sterilize raw sugar beet juice for use as a raw material in ethanol production. In the sugarcane industry, ozone is used only for the decolorization and clarification of sugar liquors [11, 19, 27]. Thermal sterilization of sugar beet juices is currently the most widely used method, but it has disadvantages. It is energy intensive and requires special equipment operating at high pressures. Moreover, in environments rich in carbohydrates and proteins, thermal processes induce Maillard reactions, generating fermentation inhibitors. Ozonation can be carried out in a less expensive flow apparatus with an ozone generator producing the gas from easily accessible oxygen. However, use of such technology on an industrial scale would require numerous studies to determine the efficacy of using ozone to sterilize media against the most common bacterial strains infecting fermentation worts. It is also important to demonstrate that ozonation does not generate fermentation inhibitors which would make the process of ethanol production less efficient.

This work studies the effectiveness of ozonation as a sterilization method on media containing raw sugar beet juice. Its influence on ethanol fermentation dynamics is also investigated. The scope of the research includes the preparation of fermentation worts from raw juice contaminated by microbial strains of typical spoilage microflora and their subjection to one of two sterilization methods: ozonation or pressure–thermal sterilization. The level of contamination was set at 10^5^ cfu/mL, because a minimum 5-log reduction of pathogens is necessary to improve sanitary processing according to HACCP regulations [35]. After sterilization of the raw sugar beet juice, ethanol fermentation was conducted and an assessment made of the indicators in the process.

## Methods

### Raw sugar beet juice

A single batch of raw sugar beet juice produced in a sugar factory (Dobrzelin Sugar Factory, Poland) through extraction from cossettes was used in this study. The fresh material was analyzed following methods recommended for the sugar industry [1]. The juice contained 16.93^o^Bx of solid substances (s.s.). Other parameters determined for the juice are listed in Table 1. The juice was stored in propylene bottles *T* = −18 °C and was naturally defrosted until it reached room temperature (20 °C) for the experiment.

**Table 1:** Composition of raw sugar beet juice

Parameter	Value
Content of solid substance (^o^Bx)	14.96
pH	5.95
Total sugars (g invert sugar/100 g)	13.39
Reducing sugars (g invert sugar/100 g)	0.99
Saccharose (g/100 g)	11.78
Total nitrogen (% *w/w*)	0.28
Volatile acids as acetic acid (% *w/w*)	0.04

### Microorganisms and inoculations

To evaluate the efficiency of the ozone sterilization process, the raw sugar beet juice was contaminated with the microbial strains *Bacillus subtilis* B01644, *Leuconostoc mesenteroides* ŁOCK 0964, *Geobacillus stearothermophilus* LOCK 0815 *Candida vini* syn. *Candida mycoderma* ŁOCK 0008, and *Aspergillus brasiliensis* ATCC16404, often isolated as microflora from spoiled raw sugar beet juice. After tyndallization (80 °C, three times), the sugar beet juice was inoculated (10^5^ cfu/mL) using 1 mL of suspension with bacterial or yeast cells or 2 mL of suspension with conidia of *A. brasiliensis*. The level of microbial contamination was verified on appropriate agar media under incubation conditions using the plate count method (Table 2).

**Table 2:** Incubation conditions with plate count method

Strain	Type of cells	Agar medium	Temperature (°C)	Time (h)
*Bacillus subtilis*	Vegetative cells and spores	PCA (Merck)	30	72
*Leuconostoc mesenteroides*	Vegetative cells	MRS (Merck)	30	72
*Geobacillus stearothermophilus*	Vegetative cells and spores	PCA (Merck)	55	72
*Candida vini*	Vegetative cells	OGY (Merck)	30	72
*Aspergillus brasiliensis*	Conidia	OGY (Merck)	28	96

Fermentation trials were conducted with Ethanol Red (*Saccharomyces cerevisiae*) (Fermentis Division S.I. Lesaffre, France).

### Sterilization of fermentation media

Before fermentation, the culture media were heat sterilized (autoclaving, 121 °C, 20 min) or ozonated (0.1 g O_3_/L O_2_ 30 min, at intervals). Ozone gas was produced with an ozone generator (Ozone Generator BMT 83 N, BMT Messtechnik, Berlin, Germany), in which ozone is produced by a corona discharge generator. Pure oxygen was supplied via an oxygen cylinder (Air Products Ltd., 99.999) and the flow rate controlled using an oxygen flow regulator. The ozone concentration was recorded using an ozone analyzer (Ozone Analyzer BMT 963, BMT Messtechnik, Berlin, Germany). Sugar beet juice samples (100 mL) were processed in a 200-mL ozone bubble column. The ozone in the oxygen stream was bubbled through with a flow rate of 6 L/h at a concentration of 0.1 g O_3_/L O_2_ in each treatment for 30 min at ambient temperature (18–20 °C). The number of viable cells able to grow on agar media was counted and their quality assessed. Before and after ozonation, in all samples, the number of colony-forming units (cfu) of microorganisms was determined and the concentration of dissolved oxygen measured using an InPro 6000 O_2_ sensor.

### Fermentation experiments

Fermentation medium was prepared from undiluted raw juice contaminated with the aforementioned microorganisms at a concentration of 10^5^ cfu/mL and then pretreated using ozonation and sterilization methods. As control samples were used: (A) fresh raw juice obtained from the sugar factory and immediately subjected to fermentation (the total number of bacteria was initially 2.5 × 10^3^ cfu/mL) and (B) the same raw juice, stored for 2 days at a temperature of 5 °C before being submitted to fermentation (the total number of bacteria was 2.0 × 10^5^ cfu/mL). Subsequently, all the worts were acidified using 25 % sulfuric acid (H_2_SO_4_, POCh SA, Poland) to pH 4.8. The salt (NH_4_)_2_HPO_4_ was added to the fermentation medium as a nitrogen and phosphorus source with a dose of 0.3 g/L. Fermentations were conducted using the dry-distillery yeast Ethanol Red (*Saccharomyces cerevisiae)* at a dose of 2 g/L. Glass fermentation flasks (2 L), each containing approximately 1 L of fermentation medium after inoculation with yeast cells, were closed with fermentation locks containing paraffin oil and kept at 28–30 °C for 48 h. Gravimetric analysis was used to measure any decrease in the mass of the worts related to the liberation of carbon dioxide (i.e., periodic measurement of the weight of the flat-bottomed flasks containing fermenting wort). Samples of the worts were collected periodically to determine their ethanol content.

### Oxygen concentration in fermentation medium

The level of dissolved oxygen in the fermentation media obtained from raw juice was monitored throughout the process of ozonation using an oxygen sensor (InPro 6000 Oxygen sensor; Mettler Toledo, Switzerland). The same measurements were also taken for water control samples.

### Analytical methods

Raw juice was analyzed following methods recommended for the sugar industry [1]. Total extract was measured using a hydrometer which indicates the concentration of dissolved solids, mostly sugars, calibrated in gram of saccharose per kilogram of water solution. The Kjeldahl method was used to determine total nitrogen. Volatile acids (expressed as acetic acid) were assayed using steam distillation. The Lane–Eynon method was used to determine the amounts of reducing sugars and total sugars (after inversion with hydrochloric acid), both expressed in gram of invert sugar per kilogram of thick juice. The saccharose concentration was calculated as the difference between total sugars and reducing sugars (with a conversion coefficient of 0.95). pH was also measured using a digital pH meter.

The media were analyzed before and after fermentation using recommended methods for distilleries. Prior to fermentation, the worts were analyzed for pH, total extract, reducing sugars (expressed as invert sugar) and saccharose content. After fermentation, the worts were analyzed for real extract (after ethanol distillation), ethanol concentration (using a hydrometer in % *v/v* of ethanol), and content of sugars.

The ethanol concentration in the distillates was assayed using refractometric measurements. Raw spirits containing around 23 % *v/v* of ethanol were refined to approximately 43 % *v/v* of ethanol and subjected to GC-FID analysis.

The distillates were analyzed using an Agilent 6890 N gas chromatograph (USA) equipped with a flame-ionization detector (FID), a split/splitless injector, and an HP-Innowax capillary column (60 m × 32 mm × 0.5 μm). The temperature was kept at 250 °C at the injector (split 1:45) and FID. The temperature program was as follows: 40 °C (6 min), increased to 83 °C (2 °C/min), and then to 190 °C (5 °C/min) (2 min). The flow rate of the carrier gas (helium) through the column was 2 mL/min.

### Statistics

For each experiment, the trials were repeated in triplicate. The results were submitted to analysis of variance (ANOVA) at a significance level of *p* < 0.05 using the Origin 7.5 software.

## Results and discussion

The parameters of the raw sugar beet juice obtained from Dobrzelin Sugar Factory were consistent with the literature [25]. The chemical composition of raw sugar beet juice is typical for this kind of material and makes it highly suitable for alcoholic fermentation (Table 1).

**Fig. 1:**
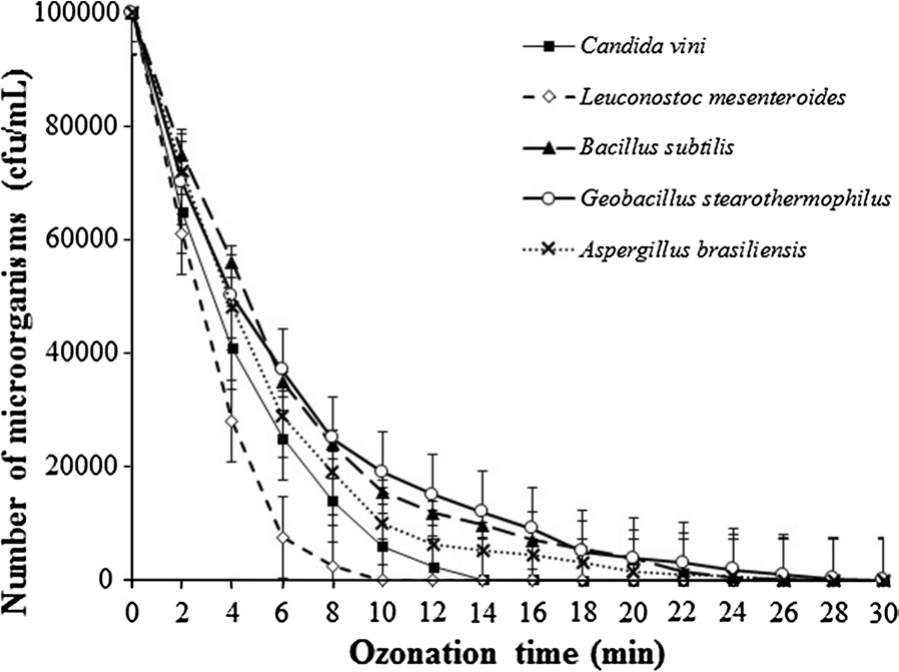
Dependence of the number of colony-forming cells of microorganisms: *Candida vini*, *Leuconostoc mesenteroides*, *Bacillus subtilis*, *Geobacillus stearothermophilus,* and *Aspergillus brasiliensisis* in raw beet sugar juice on time ozonation

One of the main factors affecting the production of ethanol from raw beet juice is its microbial instability and the possibility of infection by particular strains of bacteria and mold. To prevent infection, the juice is sterilized using various methods before fermentation. To evaluate the efficiency of the process of ozone sterilization, the raw sugar beet juice was artificially contaminated with the microbial strains *Bacillus subtilis* B01644, *Leuconostoc mesenteroides* ŁOCK 0964, *Geobacillus stearothermophilus* LOCK 0815 *Candida vini* syn. *Candida mycoderma* ŁOCK 0008, and *Aspergillus brasiliensis* ATCC16404. The conditions for incubation of microbial strains used are presented in Table 2. The initial populations of *C. vini, L. mesenteroides, B. subtilis, G. stearothermophilus,* and *Aspergillus brasiliensisis* juices were approximately 10^5^ cfu/mL of inoculated raw sugar beet (Fig. 1).

**Fig. 2:**
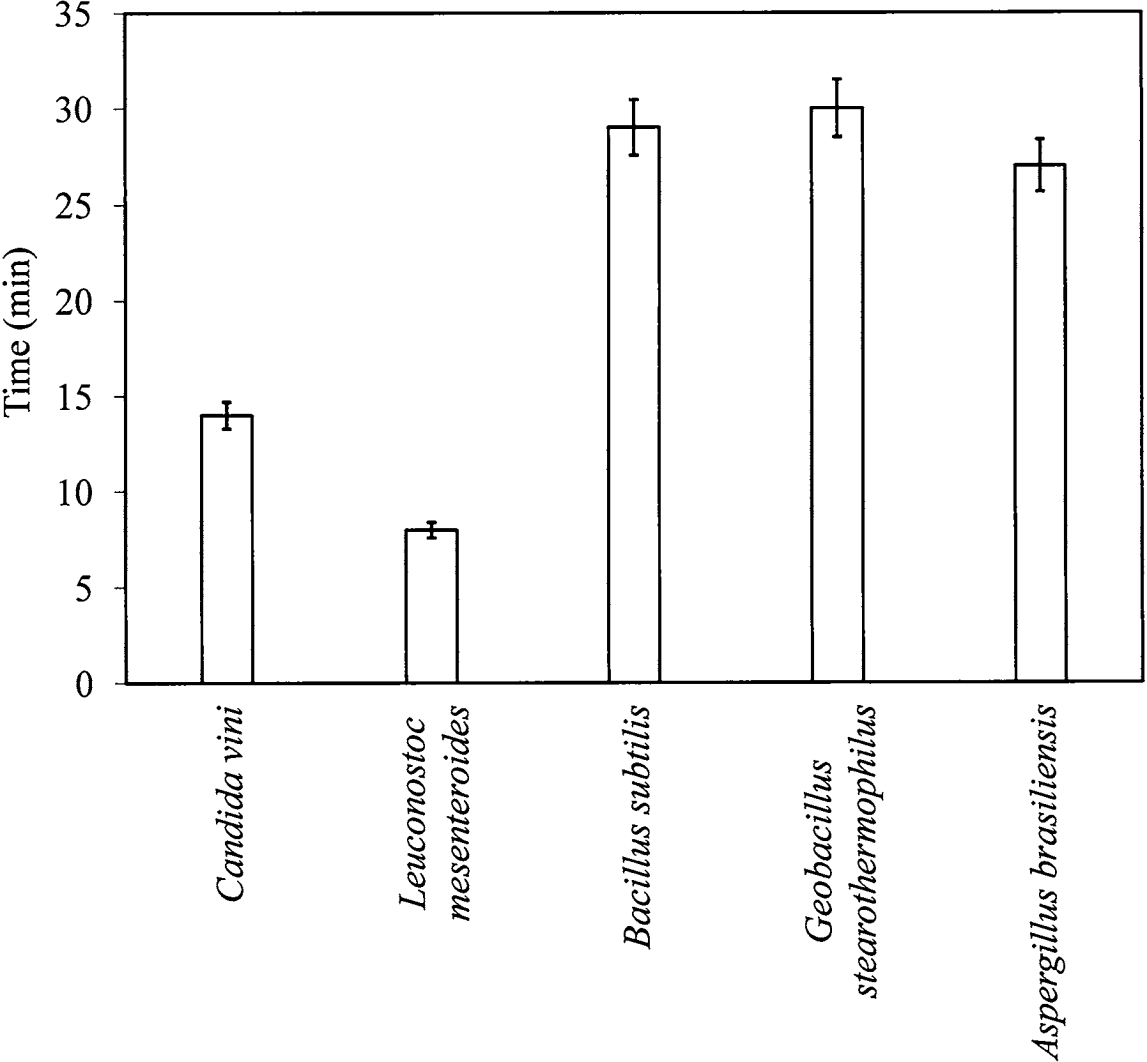
Time required for cell inactivation of *Candida vini*, *Leuconostoc mesenteroides*, *Bacillus subtilis*, *Geobacillus stearothermophilus,* and *Aspergillus brasiliensisis* in raw beet sugar juice treated with ozone

The time needed to destroy microbial cells varied, depending on the kind of microorganism and the type of cells (vegetative or spores) (Fig. 2). The vegetative cells of bacteria and yeast were killed after 8–14 min of ozone treatment, whereas spores and conidia were inactivated after 27–30 min. The time needed to sterilize raw sugar beet juice is less than that required for the sterilization of fruit juices. In a study by Sung et al. [29], apple juice was inoculated with a mixed culture cocktail (*Escherichia coli* O157: H7, *Salomonella* Typhimurium, and *Listeria monocytogenes*). The final cell concentration was 105–106 CFU/mL. The authors report the complete removal of microorganisms from the juice following ozone treatment (2–3 g O_3_/min with flow rate 3 L/min) at 50 °C for 1 h.

The results obtained for raw sugar juice support the possibility of using ozone to sterilize fermentation worts based on this material. The short length of time required to totally sterilize raw juice suggests the further opportunity of using flow ozonation technology on a large scale in the commercial production of ethanol as a fuel. Use of this technology could significantly reduce equipment costs and make bioethanol production from raw sugar beet juice more economically viable.

### Effect of different modes of sterilizing media on fermentation results

**Fig. 3:**
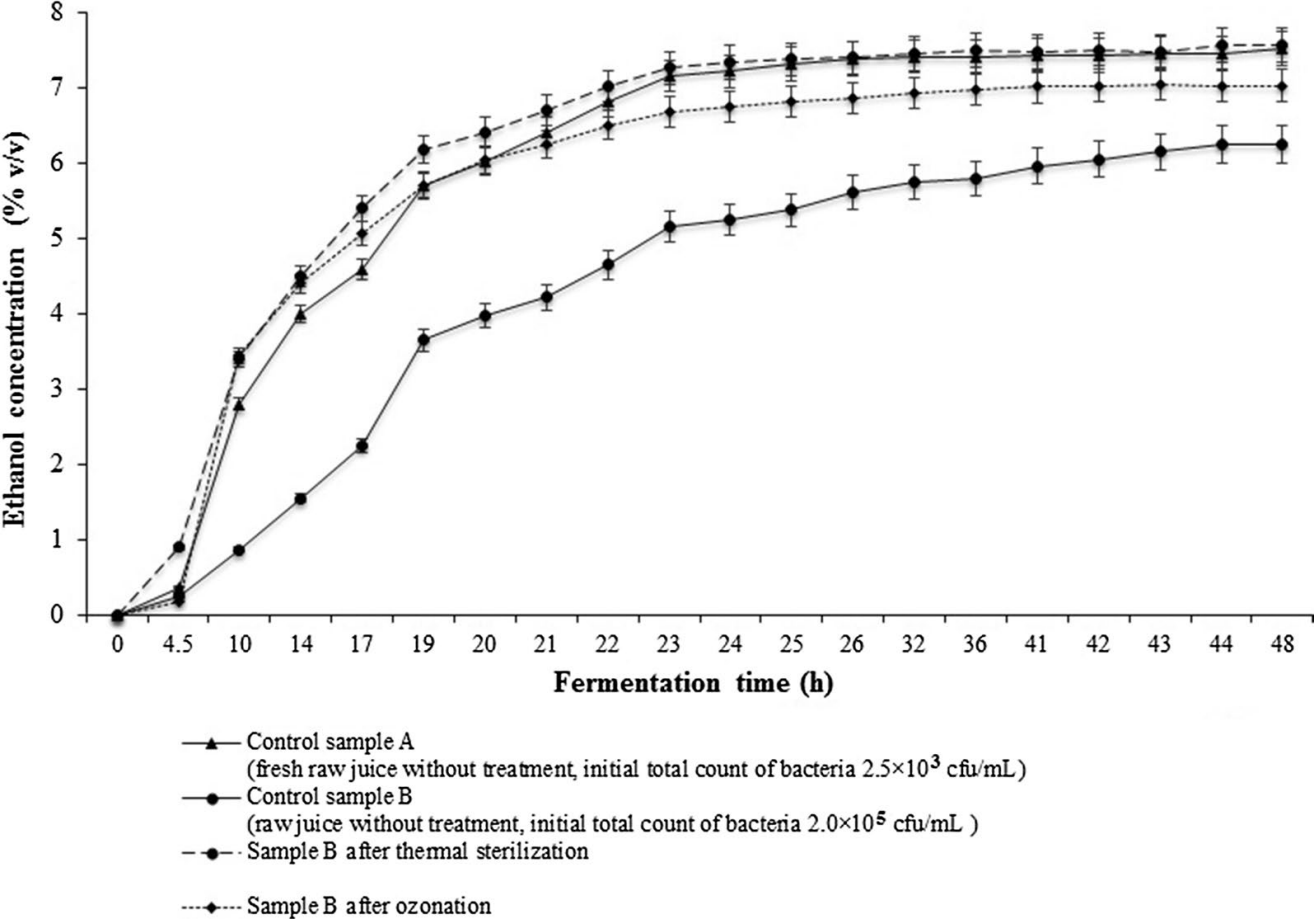
Fermentation dynamics of raw sugar beet wort (average values of three independent runs performed on untreated, thermally sterilized, and ozonated worts)

The second stage of the study investigated the effect that different modes of sterilizing the raw sugar beet juice wort had on the fermentation results. Fermentation experiments were carried out on 1 L of three kinds of sugar beet juices. The media were sterilized using autoclaving (at 121 °C, 0.1 MPa, 30 min) or ozonation (100 g O_3_/m^3^, 30 min). The control was culture medium without treatment. After fermentation, the data collected were used to plot the fermentation dynamics (Fig. 3).

In all trials, the yeast Ethanol Red fermented dynamically without a long adaptation phase. A slightly longer initial fermentation phase was observed only in media without sterilization. This short, dynamic initial phase of fermentation could be explained by the fact that thermally sterilized and ozonated wort contains more dissolved oxygen, which is needed for the initial propagation of yeast cells. Such a conclusion is supported by the oxygen content found in wort subjected to ozone (Table 3). The results show that after around 5 min of treatment with a mixture of ozone in oxygen, the concentration of dissolved oxygen in the wort more than doubled, creating conditions suitable for the initial phase of fermentation. However, the duration of the main phase of fermentation of worts pre-prepared in different ways was almost the same (ca. 24–27 h) (Fig. 3).

**Table 3:** Oxygen concentration in medium after ozonation

Medium treated with ozone	Time of ozonation (min)	Oxygen concentration in medium after ozonation (%)
Water	0	84.5
	5	127.8
	10	127.8
	15	127.8
	20	127.8
Wort obtained from raw sugar beet juice (14.96ºBx)	0	55.1
	5	127.8
	10	127.8
	15	127.8
	20	127.8

From the fermentation results in Table 4, it can be seen that pretreatment of raw sugar beet juice-based worts using thermal sterilization and ozonation had no statistically significant effect on sugar intake, ethanol production, or ethanol yield in comparison with reference wort (A) (fresh raw juice without treatment, initial total count of bacteria 2.5 × 10^3^ cfu/mL). On the other hand, comparison with control sample B, come from control sample A after 2 days incubation at 5 °C (raw juice without treatment, initial total count of bacteria 2.0 × 10^5^ cfu/mL) reveals an increase in microbial contamination of fermentation medium from 2.5 × 10^3^ to 2.0 × 10^5^ cfu/mL, suggesting that the application of thermal or ozone pretreatment may be desirable. Although the intake of sugar in control sample (B) was comparable both to control sample (A) and with trials preceded by thermal sterilization or ozonation, the ethanol concentration and yield were significantly lower. Therefore, pretreatment of raw juice (particularly with higher degrees of microbial contamination) seems necessary, especially under industrial conditions, where there is a higher risk of wort developing undesirable microflora, compared with laboratory conditions.

**Table 4:** Raw sugar beet juice wort before and after fermentation

Physicochemical parameters	Wort before fermentation			
	Control sample A (fresh raw juice without treatment, the initial total count of bacteria 2.5 × 10^3^ cfu/mL)	Control sample B (raw juice without treatment, initial total count of bacteria 2.0 × 10^5^ cfu/mL)	Sample B after	
			Sterilization	Ozonation
Dry matter (g kg^−1^)	146.0 a ± 5.0	145.0 a ± 6.0	145.0 a ± 6.0	138.0 a ± 4.0
pH	4.8 a ± 0.2	4.8 a ± 0.1	4.8 a ± 0.1	4.8 a ± 0.1
*Sugars*				
Reducing (g inverted sugar/kg)	9.4 a ± 0.3	9.7 a ± 0.3	9.3 a ± 0.4	11.7 b ± 0.4
Saccharose (g/kg)	113.7 b ± 3.4	112.5 b ± 2.4	113.1 b ± 4.5	94.8 a ± 3.8
Total (g inverted sugar/kg)	129.1 b ± 3.9	128.1 b ± 3.8	128.4 b ± 5.1	111.5 a ± 4.5

Physicochemical parameters	Wort after fermentation			
	Control sample A (fresh raw juice without treatment, initial total count of bacteria 2.5 × 10^3^ cfu/mL)	Control sample B (raw juice without treatment, the initial total count of bacteria 2.0 × 10^5^ cfu/mL)	Sample B after	
			Sterilization	Ozonation
Dry matter (g/kg)	26.5 a ± 2.0	26.2 a ± 1.5	28.2 a ± 2.1	26.1 a ± 1.0
pH	3.9 a ± 0.1	3.4 b ± 0.3	3.8 a ± 0.1	3.8 a ± 0.1
*Sugars*				
Reducing (g inverted sugar/kg)	1.9 b ± 0.1	1.4 a ± 0.1	1.6 a ± 0.1	1.9 b ± 0.2
Saccharose (g/kg)	0.5 b ± 0.1	0.3 a ± 0.1	0.2 a ± 0.1	0.6 c ± 0.0
Total (g inverted sugar/kg)	2.4 b ± 0.1	1.9 a ± 0.1	1.9 a ± 0.1	2.5 b ± 0.1
Sugar consumption (%)	98.14 a ± 2.90	98.80 a ± 2.50	98.52 a ± 3.00	98.22 a ± 2.80
Ethanol concentration (% *v/v*)	7.53 a ± 0.30	6.25 b ± 0.25	7.56 a ± 0.29	7.03 a ± 0.27
Yield of ethanol (% of theoretical yield)	90.07 a ± 2.80	75.30 b ± 3.00	90.87 a ± 2.70	97.37 b ± 3.80

All values are means of triplicate measurements ± standard deviation (SD)

a, b, c—means in rows with different letters differ significantly at *p* < 0.05

As mentioned previously, thermal sterilization is an energy-intensive process and requires special equipment. Moreover, it leads to loss of sugars and undesirable processes may occur, such as Maillard browning and caramelization [21], the products of which can inhibit fermentation. Ozone sterilization appears superior in terms both of cost and the formation of inhibitors.

Reports in the literature show that exposure to ozone degrades sucrose to glucose and fructose [2, 14, 17]. In our study (see Table 4), we observed greater reduction of sucrose content in the fermentation medium treated with ozone in comparison to unsterilized medium. This was as a result of the partial hydrolysis of sucrose to monosaccharides, which are easily utilized by yeast cells. As a consequence, the yield of ethanol, expressed as a percentage of theoretical yield, was significantly higher in the case of ozonated wort. This shows that raw sugar beet juice treated with ozone is a good medium for efficient alcoholic fermentation.

**Fig. 4:**
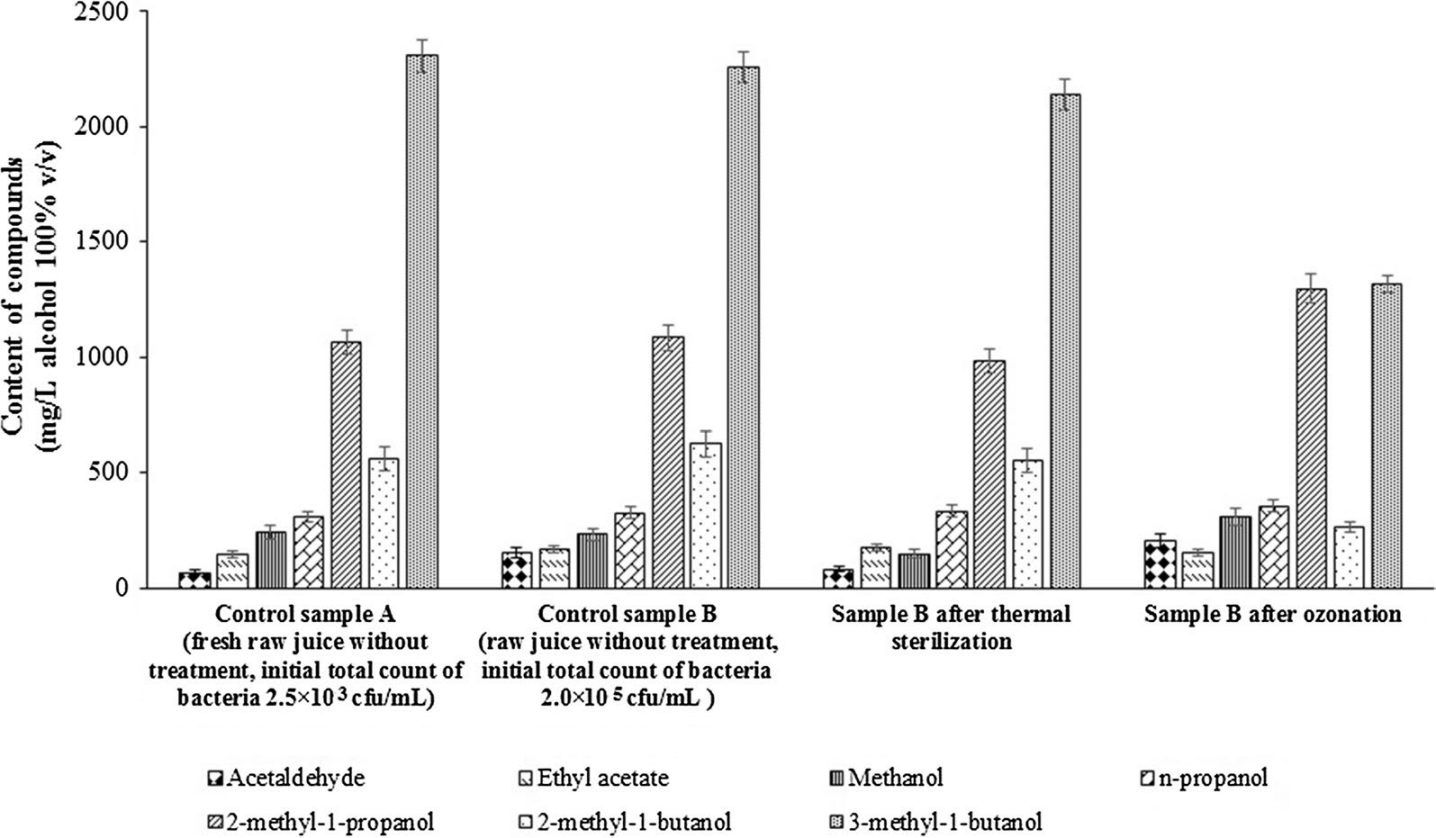
Qualitative composition and quantitative composition of major fermentation by-products in the distillates

Once the fermentation was complete (after 48 h), all the ethanol were distilled from the worts using a distillation unit and the distillates were refined to approximately 43 % *v/v* ethanol. The chemical composition of the distillates obtained from the raw sugar beet juices was determined using the GC-FID technique. The results are presented in Fig. 4.

Acetaldehyde was the main aliphatic carbonyl compound found in the tested samples of distillates from the raw juice wort. However, treatment of wort with ozone before fermentation resulted in a significant increase in the amount of this compound in the distillates (204.2 mg acetaldehyde/L alcohol 100 % *v/v*). Ozone is known to attack unsaturated bonds, forming aldehydes, ketones, and carbonyl compounds [18]. This may explain the increased level of acetaldehyde in distillates from ozonated media. Increased levels of methanol were also observed in the distillates from ozonated wort samples compared with the control samples. However, pretreatment of wort by ozonation led to the formation of lower amounts of higher alcohols (propanol and butanol).

## Conclusion

This study has shown ozonation to be an effective method of stabilizing fermentation media based on raw sugar beet juice, allowing for sterilization without inhibiting yeast cell growth. In samples sterilized using ozone, we noted a statistically significant increase in process efficiency in comparison to fermentations conducted in raw media, especially in worts with higher microbial contamination. An additional advantage is the possibility of performing ozonation and fermentation in the same fermenter or of treating fermentation media with ozone in a flow system, as the fermentation tank is being filled. Use of ozonation for the stabilization of fermentation media based on sugar beet juices has been described in Polish patent PL 210215 B1.

## Abbreviations

HACCP: Hazard analysis and critical control point
US FDA: US Food and Drug Administration
AOAC: Association of Official Analytical Chemists
